# Supplementary material for: Qualitative understandings of the persistent use of traditional contraceptive methods using the socio-ecological model among older reproductive-age women in Khulna, Bangladesh
Source: PLoS One. 2025 Oct 31;20(10):e0335543. doi: 10.1371/journal.pone.0335543 (PMC12578137; doi:10.1371/journal.pone.0335543)
Supplement: Attachment — Submitted filename: Review on TCM study Bang.docx [file pone.0335543.s001.docx]

**Methods**

1. **Sample Size and Justification**

The study conducted 10 in-depth interviews (IDIs) with women and 7 key informant interviews (KIIs) with service providers. The study relies solely on in-depth interviews (IDIs) and key informant interviews (KIIs), missing the opportunity to include focus group discussions (FGDs). This limits the depth of social and cultural insights into TCM. On the other hand, the small sample size raises concerns about saturation (whether no new themes emerged) and generalizability.

- The authors should justify why this sample size was deemed sufficient, possibly referring to qualitative research guidelines (e.g., Braun & Clarke’s thematic analysis) or prior similar studies.

2. **District Selection and Representativeness**

- The study was conducted in Khulna District, selected due to its high TCM use. However, Bangladesh has significant regional variations in healthcare access, education, and cultural norms (e.g., Sylhet has lower contraceptive use). Moreover the findings may not reflect the experiences of women in other regions (e.g., rural Sylhet or urban Dhaka). A multi-district approach could enhance transferability.

3. **Study Participants**

The study focuses on older reproductive-age women (35–49 years) to understand persistent traditional contraceptive method (TCM) use but excludes younger women (15–34 years), despite evidence showing:

- Higher unintended pregnancy rates in younger women (Noor et al., 2011; Khan et al., 2022).
- Lower modern contraceptive prevalence (MCPR) among adolescents and young women in Bangladesh (BDHS 2022).
- Greater reliance on TCM due to stigma, lack of access, or misinformation (Kamal & Islam, 2010; Kundu et al., 2022).
- In addition to this, the use of SEM examines multi-level influences, yet the study ignores a key demographic facing unique interpersonal (parental control) and institutional (youth-unfriendly services) barriers.

**Analysis**

1. **Thematic approach and use of software**

The authors followed Braun & Clarke’s framework, but more detail is needed on:

a. Coding process: Was it inductive/deductive? How were discrepancies resolved?
b. Inter-coder reliability: Were multiple researchers involved in coding to reduce bias?
c. Software use: Mention if tools like NVivo were used for transparency.

2. **Thematic Presentation of results**

The results are clearly structured using the Socio-Ecological Model (SEM), addressing factors at the individual, interpersonal, community, institutional, and policy levels. The inclusion of rich participant quotes strengthens the thematic findings, for instance, highlighting concerns such as fear of side effects and spousal opposition. However, some contradictions emerge in the narratives: while many women expressed satisfaction with Traditional and Complementary Methods (TCM), providers emphasized their high failure rates. It would be helpful to present participants’ sociodemographic characteristics and include data on intended versus unintended pregnancies, as the current results provide limited insight into prior experiences with unplanned pregnancies. Additionally, the analysis could be strengthened by exploring how variables such as education level, income, and urban or rural residence intersect with TCM use, as the current discussion remains relatively broad.

**3. Discussion**

**1. Cross-Referencing and Contextualization**

The discussion effectively links findings to prior studies (e.g., Turkey, Indonesia) but could:

- Compare more directly with Bangladeshi literature (e.g., Kamal & Islam, 2010; Hossain et al., 2018).
  - Address contrasting evidence (e.g., some studies show mothers-in-law promote modern methods).
  - Policy gaps and recommendations need to be highlighted

**Overall Review on the research:**

The study gives useful information about the use of Traditional and Complementary Methods (TCM) in Bangladesh. However, it could be made stronger by including a wider range of participants, explaining the methods in more detail, and offering clearer policy suggestions. Using the Socio-Ecological Model (SEM) works well, but the study would benefit from also looking at how different factors like gender, income, and education levels interact.
